# Leveraging whole-genome sequencing for microbial contamination tracking and risk assessment in pharmaceutical manufacturing

**DOI:** 10.3389/fmicb.2026.1807989

**Published:** 2026-04-15

**Authors:** Qiongqiong Li, Deyin Deng, Xin Dou, Xinqin Chen, Xuhua Duan, Tingzhang Wang, Minghui Song

**Affiliations:** 1 National Medical Products Administration Innovation Laboratory for Drug Regulatory Science, Shanghai Quality Inspection and Testing Center of Innovative Biological Products, Shanghai Institute for Drug Control, Shanghai, China; 2 Key Laboratory of Microbiol Technology and Bioinformatics of Zhejiang Province, Zhejiang Institute of Microbiology, Hangzhou, China; 3 Shanghai Institute of Quality Inspection and Technical Research Co., Ltd., Shanghai, China

**Keywords:** antimicrobial resistance, contamination source tracing, horizontal gene transfer, pharmaceutical environments, virulence factors, whole-genome sequencing

## Abstract

**Background:**

Pharmaceutical cleanrooms remain vulnerable to microbial contamination introduced through environmental and personnel-associated pathways, yet the genomic characteristics and transmission dynamics of these contaminants are not well defined.

**Methods:**

In this study, whole-genome sequencing (WGS) was applied to characterize microbial populations across a sterile vaccine production line. We adopted an integrated strategy that combined routine environmental monitoring with targeted genomic investigation, mapping the overall distribution of microorganisms and prioritizing isolates showing abnormal or recurrent detection for sequencing. By integrating SNP-based phylogeny, sampling location metadata, and ARG/virulence profiling. we identified five dominant opportunistic species, each displaying distinct genomic signatures indicative of different introduction pathways.

**Results:**

By combining species-specific genomic features with their sampling distributions, we reconstructed four plausible contamination chains: clonal inward dissemination of *Burkholderia contaminans* from Grade B to Grade A areas, repeated water-associated introductions of *Ralstonia pickettii*, intermittent personnel-mediated seeding of *Staphylococcus epidermidis*, and sporadic external incursions of *Microbacterium laevaniformans*. ARGs and VFs were heterogeneously distributed, with mobile resistance determinants and colonization- or immune-evasion factors concentrated in *B. contaminans* and *R. pickettii*, suggesting elevated persistence potential under disinfectant pressure.

**Conclusion:**

These findings demonstrate that WGS enables precise source attribution and transmission-route reconstruction beyond the capability of conventional typing methods, thereby supporting real-time contamination control and evidence-based microbial-risk management in pharmaceutical manufacturing.

## 1 Introduction

Microbial contamination represents a critical vulnerability in pharmaceutical manufacturing, particularly for sterile products where even low-level environmental intrusions can compromise product safety and trigger life-threatening infections. Between 2012 and 2019, over 50% of drug product recalls reported by the U.S. Food and Drug Administration (FDA) were linked to microbiological incidents (Santos et al., 2018; Jimenez, 2019), demonstrating that microbial intrusion remains a persistent challenge in modern pharmaceutical critical manufacturing zones. Effective microbial environmental monitoring is therefore fundamental to maintaining compliance with current good manufacturing practices (cGMP) and ensuring product quality (Kawai et al., 2019). Numerous investigations and regulatory alerts have emphasized the strong association between the level of environmental control and the microbiological quality of final products (Jimenez, 2007; Sandle, 2012; Jain and Jain, 2018).

Understanding the composition and dynamics of environmental microflora in pharmaceutical manufacturing facilities is fundamental to the development of effective contamination control strategies (Hamdy et al., 2018). Characterizing the resident microbial population not only supports the identification of contamination sources but also provides valuable indicators for evaluating disinfection efficacy and the robustness of environmental monitoring programs (Akers, 1997; Wu and Liu, 2007; Halls, 2004). However, current studies on pharmaceutical cleanroom microbiota remain limited in both scope and resolution (Wu and Liu, 2007; Sandle, 2011; Vijayakumar et al., 2012; Park et al., 2014; Vijayakumar et al., 2016), relying predominantly on culture-dependent or 16S rRNA-based methods that rarely resolve below the genus level and are intrinsically blind to strain-level diversity as well as horizontally acquired resistance and virulence determinants (Ranjan et al., 2016).

Early detection of strain transmission events is equally critical for preventing microbial spread and enabling timely corrective actions. Conventional molecular typing methods such as pulsed-field gel electrophoresis (PFGE) and multilocus sequence typing (MLST) have contributed to contamination tracing and epidemiological linkage analysis (Simar et al., 2021), yet their discriminatory power and scalability are limited. In contrast, whole-genome sequencing (WGS) provides unprecedented strain-level resolution, enabling precise source attribution, outbreak investigation, and comprehensive profiling of antimicrobial resistance genes (ARGs) and virulence factors (VFs) (Miro et al., 2019; Uelze et al., 2020; Menghwar et al., 2022). Single-nucleotide polymorphism (SNP)-based comparative genomics, in particular, has emerged as a powerful approach for differentiating closely related isolates and uncovering potential transmission routes across pharmaceutical production environments.

Beyond strain-level source tracing, the genomic characterization of antimicrobial resistance and virulence determinants is essential for assessing the functional risks of microbial persistence in manufacturing facilities. Antimicrobial resistance (AMR) in pathogenic bacteria constitutes a considerable public-health concern (Baker-Austin et al., 2006). Despite tremendous efforts to restrict usage of several key antibiotics worldwide, AMR is frequently detected in different environments (Li et al., 2015; Rodriguez-Mozaz et al., 2015). Generally, the occurrence of AMR is largely driven by the selection pressure of antibiotics (Aarestrup, 2012). Besides that, other agents such as antibacterial biocides can help to maintain AMR via co-selection (Ashbolt et al., 2013; Ding et al., 2019; Mazhar et al., 2021). For example, some studies have shown that excessive use of quaternary ammonium compounds (QACs) can select for bacteria with reduced susceptibility to antibiotics (Buffet-Bataillon et al., 2012; Tandukar et al., 2013). In pharmaceutical production environments where large quantities of disinfectants and selective antimicrobial agents are routinely applied, the resulting selection pressure may foster the enrichment and persistence of resistant populations, thereby accelerating the dissemination of ARGs through horizontal gene transfer. The co-localization of ARGs with mobile genetic elements (MGEs) further amplifies their potential for rapid dissemination (Yu et al., 2016; Zhao et al., 2019), while the concurrent presence of VFs may enhance bacterial adaptability and pathogenic potential. Investigating the genomic context of ARGs and VFs in environmental isolates therefore provides critical insights into both the evolutionary mechanisms underpinning microbial survival under strong selective pressures and the potential pathways for contamination persistence and recurrence in pharmaceutical settings.

Collectively, these attributes underscore the value of WGS as an integrated framework for microbial source tracking, resistance monitoring, and ecological risk evaluation in pharmaceutical environments. The growing prevalence of antimicrobial-resistance bacteria (ARB) further highlights the urgency of comprehensive microbial risk surveillance. The emergence of ARB strains severely restricts therapeutic options and compromises the efficacy of antimicrobial treatments, leading to increased morbidity, mortality, and healthcare costs (Coast et al., 1996; Smith and Coast, 2002). In this context, personnel working in pharmaceutical manufacturing facilities may face a heightened risk of exposure to resistant microorganisms, particularly in settings where antibiotics and disinfectants are extensively used. These occupational exposures could potentially contribute to colonization or infection by resistant strains already enriched in the production environment. Although extensive work has been conducted in clinical and agricultural contexts (Ji et al., 2012; Biswas et al., 2019; Li et al., 2022), investigations of antimicrobial resistance in pharmaceutical production settings remain scarce.

In the study, a combination of Bruker MALDI-TOF MS and 16S rRNA gene sequencing was used to classify isolates recovered from the environmental monitoring system spanning the entire production workflow, allowing us to characterize the dominant microbial communities present in the environment. To further examine the microbial composition, resistance profiles, and virulence factors (VFs) within the manufacturing setting, WGS was performed on 38 abnormal samples collected across the sterile manufacturing line. By applying SNP-based tracing, we achieved a detailed assessment of microbial distribution, contamination patterns, and transmission pathways. Integrating comprehensive bioinformatics approaches, we explored the dissemination routes of ARGs and mapped the distribution of VFs across the production line, examining the relationships among VF types, categories, and their hosts. This work aimed to provide a framework for the pharmaceutical industry to understand microbial risks throughout the production process and demonstrates the potential of WGS for monitoring ARGs, VFs, and their transmission in specialized manufacturing environments.

## 2 Materials and methods

### 2.1 Environmental monitoring plan and sampling

From 2022–2023, an environmental monitoring program was performed in sterile pharmaceutical manufacturing facility in Shanghai, China. Sampling covered two main production areas, drug product (DP) and drug substance (DS) manufacturing, both operated under four cleanroom grades. Samples were collected from different parts of the manufacturing process, including finished products, raw materials, personnel, and environmental surfaces.

Several pharmacopeial methods were used: (a) active air sampling with a volumetric air sampler; (b) settle plates for passive air collection; (c) swabs for irregular surfaces; (d) contact plates for flat surfaces; and (e) 100-mL membrane filtration onto Reasoner’s 2A agar (R2A, bioMérieux, France) for water for injection. Trypticase Soy Agar (TSA, bioMérieux, France) was used for all other sample types, with neutralizers added when disinfectant residues were expected.

A relatively intensive sampling schedule was applied, and the frequency for each monitoring activity was determined based on the assessed microbiological risk. In general, settle plates were exposed throughout operations and replaced every 4 h in Grade A and B rooms. Active air samples were taken once per shift. In Grade C areas, both active and passive air samples were taken for each batch. Hard surfaces, such as filling equipment and product contact parts, were sampled every shift in Grade A and B, and weekly in Grade C. All collected plates were incubated aerobically at 30 ~ 35 °C for 3 ~ 5 days. Detailed sample information was provided in Supplementary Table S1.

All sampling sites were selected based on comprehensive risk assessment to represent potential contamination sources throughout the entire production workflow. Sampling procedures followed standardized environmental monitoring protocols in compliance with cGMP regulations for cleanroom validation.

### 2.2 MALDI-TOF MS identification

This study employed an on-target extraction approach to support rapid and standardized species identification. In brief, a single fresh colony was transferred onto a polished steel MSP 384 target plate (Bruker Daltonik GmbH, Germany). One microliter of 70% formic acid was applied to the smear and allowed to dry, after which 1 μL of a-cyano-4-hydroxycinnamic acid (HCCA) matrix solution (10 mg/mL in 50% acetonitrile, 2.5% trifluoroacetic acid, and 47.5% water) was added and dried again. Mass spectra were acquired using an Autoflex TOF/TOF mass spectrometer (Bruker Daltonik GmbH, Germany) operated in linear positive mode. Identification was performed with the MALDI Biotyper Compass software (version Compass) using the 7,854-entry reference library and default settings. Calibration was conducted with the Bruker Bacterial Test Standard (BTS) according to the manufacturer’s protocol. Each isolate was analyzed in duplicate, and the result with the higher log score was used.

### 2.3 16S rRNA gene sequencing analysis

Isolates retained after purification were grown on TSA at 35 °C for 24 h prior to molecular identification. Genomic DNA was extracted using the Takara DNA extraction kit (Takara Bio Inc., Shiga, Japan) following the supplier’s instructions. The nearly full-length 16S rRNA gene was amplified using the universal primers 27F (5′-AGTTTGATCMTGGCTCAG-3′) and 1492R (5′-GGTTACCTTGTTACGACTT-3′). PCR products were verified on 1.2% agarose gels stained with ethidium bromide (0.5 μg/mL). Bidirectional sequencing was performed by a commercial provider (Sangon Co., Ltd., China). Sequence chromatograms were assembled and analyzed using Lasergene 7.0 (DNAStar, Madison, WI).

### 2.4 Sample collection and processing for WGS

We conducted WGS of abnormal environmental samples collected from 38 distinct sites across the sterile pharmaceutical manufacturing facility. Sampling locations encompassed critical functional areas, including bulk liquid preparation zones (e.g., bulk liquid containers at various production stages, sampled from both bottom and surface regions within Grade B and D cleanroom environments), purification systems [e.g., tryptic soy broth (TSB) simulation units and freeze-drying systems used for process validation], cleanroom surfaces (ISO Grade B and D, covering key equipment interfaces and environmental control points), utility systems (e.g., sinks, water baths, and collection tanks), personnel contact sites (e.g., clothing, skin, and respiratory protective equipment), and airborne monitoring sites (e.g., active air sampling for viable microorganisms). Detailed information regarding the sampling sites and facility characteristics is provided in Supplementary Table S2.

### 2.5 DNA extraction and sequencing for WGS

Microbial isolates were streaked onto TSA plates and incubated at 37 °C for 24 h to ensure culture activation. Following incubation, appropriate amounts of bacterial biomass were collected, and genomic DNA was extracted using the Extraction Kit v3.0 (Takara Bio Inc., Shiga, Japan) according to the manufacturer’s instructions. The concentration and purity of extracted DNA were determined using a fluorescence-based nucleic acid quantification system and corresponding reagents. Only samples with DNA concentrations exceeding 10 ng/μL, meeting the quality requirements for microbial whole-genome library construction, were retained for sequencing.

For the 38 target microbial isolates included in this study, WGS libraries were prepared using the *Nextera™ DNA Flex Library Preparation Kit* (Illumina, United States) following the manufacturer’s protocol. The constructed libraries were then subjected to high-throughput sequencing on an Illumina sequencing platform in accordance with standard operational procedures.

### 2.6 *De novo* quality controls and assembly

The quality metrics of whole-genome sequencing data for the 38 microbial isolates are summarized in Supplementary Table S3. All samples exhibited Q30 values exceeding 95%, indicating high-quality sequencing data with sufficient depth to comprehensively cover the genomic information of each isolate.

Raw sequencing reads were processed using fastp (Chen et al., 2018) to perform quality control, including the removal of low-quality reads (Phred score < 20), reads containing a high proportion of ambiguous bases (N), and adapter trimming. The resulting clean reads were subsequently assembled using Unicycler (Wick et al., 2017), generating high-quality draft genomes for all isolates. Genome completeness and contamination were assessed using CheckM2 (v1.1.0) with default parameters. Assembly statistics, including total assembly size, N50 value, and GC content, as well as completeness and contamination estimates, were obtained from the CheckM2 output and are summarized in Supplementary Table S3. These results demonstrate that reliable genome assemblies were obtained for all 38 strains.

### 2.7 Average nucleotide identity calculations and species identification

Based on the assembled contig sequences, taxonomic classification was performed using GTDB-Tk (v2.3.0) with the classify_wf workflow against the Genome Taxonomy Database (GTDB) (Letunic and Bork, 2024) reference database to assign taxonomic identities. FastANI (v1.33) (Goris et al., 2007) was employed to calculate pairwise average nucleotide identity (ANI) values between the isolates and reference genomes.

A species delineation threshold of 95.0% ANI was applied, in accordance with current genomic taxonomy standards, to determine species-level relationships among the isolates. Based on genomic similarity results, the taxonomic assignments for all 38 isolates are summarized in Supplementary Table S4. The results revealed that the isolates were distributed across five species, including *R. pickettii*, *M. laevaniformans*, *B. contaminans*, *S. epidermidis*, and *S. capitis*.

### 2.8 SNP identification and phylogenetic analysis

Single-nucleotide polymorphism (SNP) calling was performed using Snippy (v4.6.0) (https://github.com/tseemann/snippy) which identifies variants relative to the reference genome and generates a core SNP alignment by excluding regions not shared among all isolates. Pairwise SNP differences between isolates were calculated based on the core SNP alignment, and the resulting distance matrix was visualized as a heatmap using the pheatmap R package to illustrate genetic relatedness. The Reference genome used in each comparison was selected as the earliest detected sample within the species. IQ-TREE2 (v2.3.0) (Minh et al., 2020) was employed to infer phylogenetic relationships based on the core SNP alignment. The best-fit substitution model was determined using the -m MFP option, and a maximum likelihood (ML) analysis was conducted with 1,000 bootstrap replicates to assess branch support. Phylogenetic trees were visualized and annotated using the ggtree R package (v3.6.2) (Xu et al., 2022). To evaluate the overlap of core SNP positions among isolates within each species, UpSet plots were generated using the UpSetR R package (v1.4.0).

### 2.9 Identification of ARGs and MGE predictions

The comprehensive antibiotic resistance database (CARD database, version 3.0.0) (Alcock et al., 2023) was used to detect ARGs. Only ARGs detected with at least 80% identity and a maximum E-value of 10–3 were retained for further analyses. ARGs and MGEs on a contig were annotated by BLASTX using SARG (Yin et al., 2018), ICEberg (Bi et al., 2012), Isfinder (Siguier et al., 2006), and IntegronFinder (Néron et al., 2022) databases. The cut-off values for a positive BLASTX hit were identity ≥70%, alignment length ≥ of 25 amino acids, and e-value ≤ 10^−4^. Contigs with length over 2 kb were used for identifying host range and genetic location of ARGs.

The genetic location of each ARG was predicted using PlasFlow (v1.1.0) (Krawczyk et al., 2018) on ARG-carrying contigs to determine whether these contigs were likely derived from plasmid or chromosomal sequences. We divided ARGs into three groups to indicate their potential mobility. 1) Mobile ARG: the ARG is found on plasmid or flanked by MGEs within 2 kb distance; 2) Immobile ARG: the ARG is found on chromosome and no MGE is observed within its 2 kb flanking region; 3) Unclassified: the genetic location of the ARG is unclassified, and the ARG is not flanked by MGEs within 2 kb.

### 2.10 Identification and annotation of virulence factors

Virulence factors in each sample were identified using the “-draft” workflow of the MetaVF toolkit (Dong et al., 2024). A sequence was considered a virulence gene when it showed >80% identity and >70% coverage to a gene in the VFDB. Each virulence factor was further annotated for VF category, bacterial host species, and mobility based on the annotation dataset of VFDB 2.0 (Dong et al., 2024).

## 3 Results

### 3.1 The abundance of microorganisms at different processing locations

**Figure 1:**
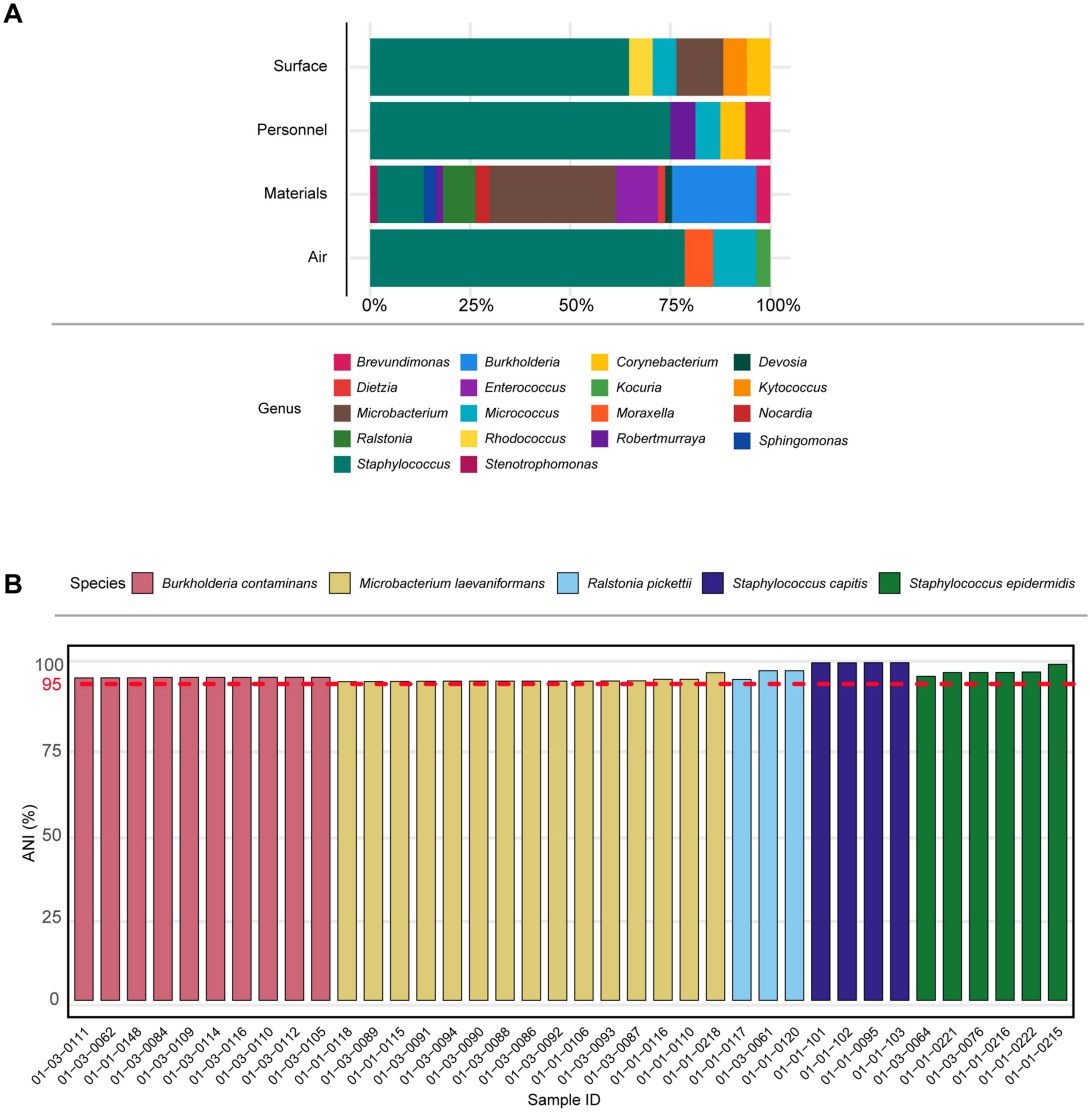
**(A)** The distribution of the predominant genus in different monitoring locations. **(B)** Comparison of average nucleotide identity (ANI) across isolates recovered from environmental samples. Species were determined based on ANI similarity to reference genomes, with a cutoff of 95% (red dashed line). Bars are colored according to species assignment: *Burkholderia contaminans* (red), *Microbacterium laevaniformans* (yellow), *Ralstonia pickettii* (light blue), *Staphylococcus capitis* (dark blue), and *Staphylococcus epidermidis* (green). All isolates above the 95% ANI threshold were considered to belong to the same species.

From 2022–2023, a total of 119 isolates were recovered from the sterile manufacturing process, including air, materials, personnel, and environmental surfaces (Figure 1A). Materials monitoring accounted for the largest proportion, with 58 isolates, followed by airborne (28 isolates), surface samples (17 isolates), and personnel monitoring (16 isolates). Genus-level identification based on MALDI-TOF MS revealed pronounced differences in microbial composition across the monitored locations (Figure 1A), with all isolates assigned to a total of 19 bacterial genera.

In environmental surface samples, the recovered isolates were dominated by *Staphylococcus*, followed by *Microbacterium*. In addition, *Corynebacterium*, *Micrococcus*, *Kytococcus*, and *Rhodococcus* were also identified, although their relative abundances were low. In personnel samples, the genus-level composition of isolates was highly concentrated, with *Staphylococcus* representing the dominant genus. Other genera, including *Robertmurraya*, *Micrococcus*, *Corynebacterium*, and *Brevundimonas*, were detected at low relative abundances. In material-associated samples, the recovered isolates exhibited a higher degree of genus-level diversity. The microbial community was primarily composed of *Microbacterium* and *Burkholderia*. In addition, several other genera, including *Stenotrophomonas*, *Sphingomonas*, *Robertmurraya*, *Ralstonia*, *Dietzia*, *Nocardia*, *Enterococcus*, *Brevundimonas*, and *Devosia*, were detected at lower relative abundances. In air samples, the genus-level composition of recovered isolates was relatively simple. *Staphylococcus* was the dominant genus, followed by *Moraxella* and *Micrococcus*. *Kocuria* was also detected at low relative abundance.

### 3.2 Genome assembly and average nucleotide identity (ANI) analysis

To identify microbiological events of potential concern, isolates showing abnormal occurrence, unexpected taxonomic identity, or recurrent detection across multiple monitoring points were prioritized for WGS. WGS was performed on the abnormal samples collected from 38 distinct sites across the sterile pharmaceutical production facilities, enabling more detailed downstream analyses. Sampling locations encompassed critical functional areas, including bulk liquid preparation zones, purification systems, cleanroom surfaces, utility systems, personnel contact sites, and airborne monitoring sites. Detailed information regarding sampling locations and facility characteristics is provided in Supplementary Table S2.

High-throughput WGS was conducted on a part of isolates, yielding consistently high-quality assemblies (Supplementary Table S3). On average, each sample produced 7,414,389 raw reads, corresponding to 1.10 Gb of sequencing data, with a mean Q30 of 96%. The assemblies demonstrated robust completeness, with an average N50 of 229,847 bp, a total genome length of 4.54 Mb, and a GC content of 57%.

Average nucleotide identity (ANI) analysis was employed to assess genetic relatedness among the assembled genomes (Figure 1B). The ANI values ranged from 95.0 to 100.0%, with an overall mean of 95.82%, confirming species-level clustering of the isolates. Consistent with established genomic taxonomy thresholds, 99.8% of the total eight billion genome pairs conformed to >95% intra-species and <83% inter-species ANI values (Jain et al., 2018), supporting the robustness of the observed species demarcation. Five dominant bacterial taxa were identified: *R. pickettii*, *M. laevaniformans*, *B. contaminans*, *S. epidermidis*, and *S. capitis*. Among these, *B. contaminans* and *M. laevaniformans* were most prevalent, whereas *Ralstonia* and *Staphylococcus* species were mainly recovered from downstream or personnel-associated sampling points.

### 3.3 Comprehensive profiling of SNP distances between each sequenced isolate of species

**Figure 2:**
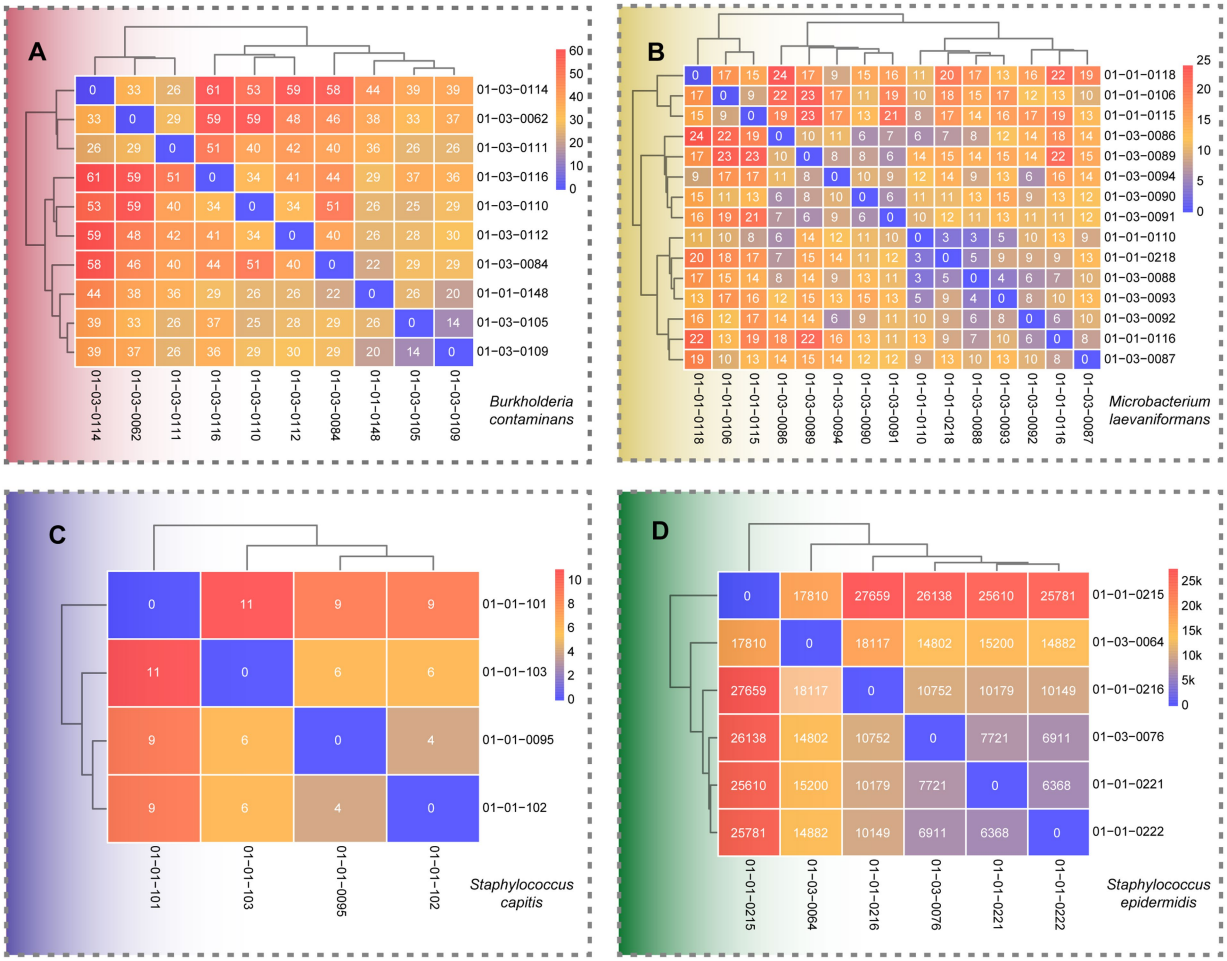
Heatmaps of pairwise SNP differences among isolates of four bacterial species. Brighter colors indicate higher SNP counts, with exact values shown in each cell. Rows and columns represent isolates, with hierarchical clustering based on SNP distances. **(A)** *Burkholderia contaminans*; **(B)** *Microbacterium laevaniformans*; **(C)** *Staphylococcus capitis*; **(D)** *Staphylococcus epidermidis*.

To assess fine-scale genomic relatedness and population structure among the sequenced isolates, pairwise SNP distance analyses were performed for each dominant species recovered from the production environment. Overall, distinct SNP distance patterns were observed across species, ranging from highly clonal populations with minimal genomic variation to genetically diverse populations comprising multiple divergent lineages (Figure 2; Supplementary Figure S1).

For *B. contaminans* isolates (Figure 2A), the pairwise SNP differences ranged from 0 to 61, with an overall tightly clustered distribution pattern. Several isolates, such as 01-03-0110, 01-03-0112, and 01-03-0108, exhibited fewer than 20 SNPs relative to one another, suggesting a close genetic relationship and potential derivation from a common ancestor or a persistent environmental source. Conversely, isolates 01-03-0114 and 01-03-0102 displayed higher SNP distances (>50), implying the presence of distinct sublineages within the same facility. The clustering pattern indicates limited genomic diversification among *B. contaminans* strains, consistent with their known adaptability and persistence in moist, nutrient-limited cleanroom environments.

Similarly, *M. laevaniformans* isolates (Figure 2B) exhibited SNP differences ranging from 0 to 24, revealing an extremely close genomic relationship among all strains. Most isolate pairs showed SNP distances clustered between 10 and 20, and hierarchical clustering revealed no clearly separated distant branches, with all isolates forming a tightly grouped genetic cluster and no evident subpopulation structure. In addition, several isolate pairs differed by only 0–5 SNPs, suggesting that they belong to the same or very closely related clonal lineages.

For *S. capitis* isolates (Figure 2C), the pairwise SNP distances ranged from 0 to 11, revealing an extremely close genomic relationship among all strains. Most isolate pairs differed by fewer than 10 SNPs, and hierarchical clustering showed that all isolates grouped tightly together without forming distinct subclusters. The minimal SNP variation indicates that the *S. capitis* population circulating in the production environment likely originated from a single clonal lineage.

In contrast, *S. epidermidis* isolates (Figure 2D), exhibited much higher genetic diversity, with SNP differences ranging from approximately 6,000 to 27,000. Hierarchical clustering revealed two and more groups of related isolates, indicating the coexistence of distinct lineages within the same production environment. The wide SNP range suggests that these isolates did not arise from a single clonal expansion but rather represent multiple genetically distinct *S. epidermidis* populations co-occurring in the facility. This observed diversity may be influenced by the broader sampling coverage across multiple locations, thereby capturing isolates with substantially divergent genetic backgrounds.

For *R. pickettii* (Supplementary Figure S1). The isolates 01-01-0120 and 01-03-0061 differ by only two SNPs, indicating that they likely represent the same transmission lineage. In contrast, isolate 01-01-0117 differs from them by approximately 190,000 SNPs, demonstrating substantial genetic divergence and suggesting it originated from a distinct source. During the phylogenetic and gene-set analyses of *R. pickettii*, attempts to construct a core-genome phylogenetic tree and generate an Upset plot did not yield informative results. As shown in the SNP heatmap (Supplementary Figure S1), several isolates exhibited extremely low genomic divergence, resulting in an insufficient number of core-gene variable sites to support a reliable phylogeny. In addition, the gene-family presence/absence profiles were highly uniform across isolates, providing no discriminative variation for meaningful Upset visualization. Therefore, no phylogenetic tree or Upset plot is presented for this species.

### 3.4 Contamination source tracking of every species using the SNP phylogenetic trees

**Figure 3:**
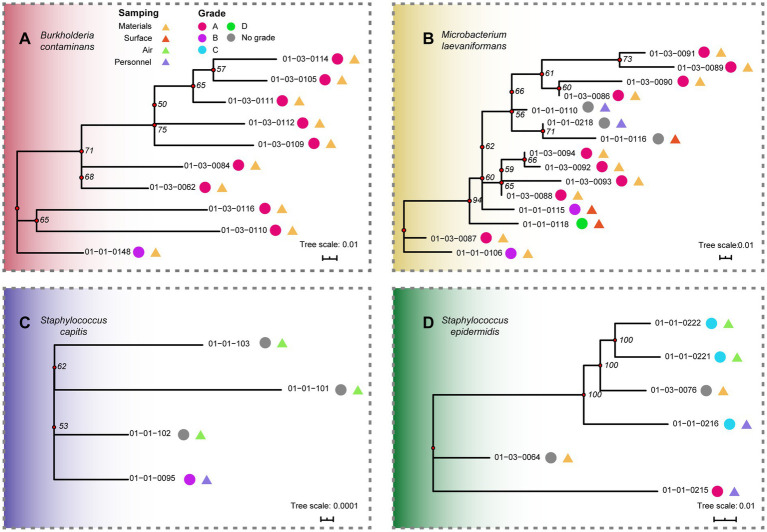
Phylogenetic trees among isolates of four bacterial species based on SNP differences. Branch lengths are proportional to genetic distance, with bootstrap values indicated at nodes. Sample labels include annotations for cleanroom grade and sampling location. **(A)** *Burkholderia contaminans*; **(B)** *Microbacterium laevaniformans*; **(C)** *Staphylococcus capitis*; **(D)** *Staphylococcus epidermidis*.

To elucidate the genetic relatedness and potential dissemination routes of dominant environmental isolates identified in the sterile pharmaceutical facility, maximum-likelihood phylogenetic trees were constructed based on core-genome SNP alignments for *B. contaminans*, *M. laevaniformans*, *S. capitis*, and *S. epidermidis* (Figures 3A–D). Overall, the four species showed distinct phylogenetic patterns, ranging from compact, low-divergence lineages to strongly separated clades, consistent with the SNP distance profiles in Figure 2.

For *B. contaminans*, all isolates formed a tight monophyletic clade with short branch lengths and strong bootstrap support (Figure 3A), indicating a high degree of genomic relatedness. Isolate 01-01-0148, recovered from F-3-047 collection liquid 2, was collected during the earliest sampling on January 1st and occupied a basal position in the phylogenetic tree. Notably, most *B. contaminans* isolates were recovered from Grade A cleanroom zones, whereas isolate 01-01-0148 originated from a Grade B environment.

The *M. laevaniformans* phylogeny (Figure 3B) revealed a single compact lineage encompassing all isolates, with limited subclade diversification, consistent with the highly clonal pattern inferred from SNP distance analysis. Considering both the isolation sources and collection timeline, the genetic relatedness among isolates obtained from the bottom surface of a Grade B material transfer bottle (01-01-0106), an electric thermostatic water bath (01-01-0118), and multiple TSB simulation samples may indicate recurrent or continuous contamination events across distinct operational units. These findings are consistent with the possibility that *M. laevaniformans* may persist within the manufacturing environment and potentially form an environmental reservoir contributing to its long-term presence.

Similarly, *S. capitis* isolates (Figure 3C) showed extremely low genetic variability and short inter-branch distances, with all isolates forming a single phylogenetic lineage. Notably, the basal isolate 01-01-0095, recovered from a Grade B personnel most base swab, occupied the deepest branching position in the phylogeny, whereas all remaining isolates originated from non-graded airborne samples. This phylogenetic structure raises the possibility that the Grade B personnel-associated isolate represents an early detected lineage that may be related to subsequent detections in the surrounding areas via airborne routes.

In contrast, *S. epidermidis* isolates (Figure 3D) displayed a distinct phylogenetic structure, separating into two well-supported clades with long branch lengths between them. Within each clade, isolates clustered closely with strong bootstrap support, indicating stable lineage structure, whereas the pronounced separation between clades suggests the presence of genetically unrelated populations. Notably, isolates from different clades were recovered from diverse sampling types and cleanroom grades, and no clear temporal or spatial progression was observed across the phylogeny. Pattern is not consistent with the long-term persistence of a single clone and instead suggests the possibility of repeated or independent introductions of *S. epidermidis* into the production environment.

### 3.5 Genomic intersection analysis of SNP

**Figure 4:**
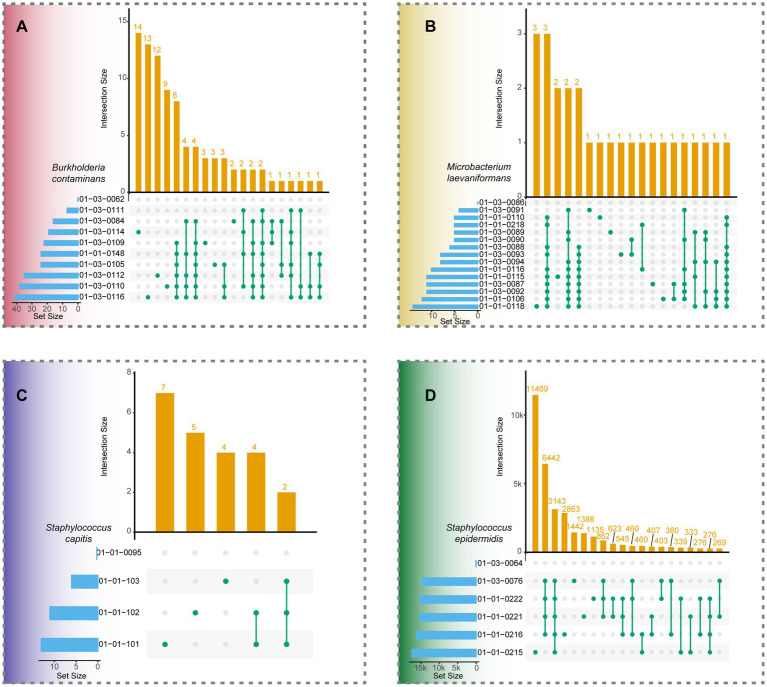
UpSet plots of the intersection of SNPs among bacterial isolates across four species. Each panel represents one species: **(A)** *Burkholderia contaminans*, **(B)** *Microbacterium laevaniformans*, **(C)** *Staphylococcus capitis, an*d **(D)** *Staphylococcus epidermidis. Th*e horizontal bars (bottom left) indicate the number of SNPs (Set Size) detected in each isolate, while the vertical bars (top) represent the number of shared SNPs (Intersection Size) among combinations of isolates. Dots connected by lines denote shared SNP sets between isolates. In each panel, the first isolate (with Set Size = 0) represents the reference genome used for SNP comparison.

Comparative genome analysis based on core SNP intersections further supported the phylogenetic findings (Figures 4A–D). For each species, the intersection plots summarized the shared and unique variant compositions among isolates relative to their respective reference genomes, which were selected as the earliest detected isolate within each species. In *B. contaminans* (Figure 4A), extensive SNP sharing was observed among isolates, with most combinations involving multiple strains. *M. laevaniformans* (Figure 4B) exhibited fewer shared SNP subsets, primarily involving one or two isolates. The *S. capitis* pattern (Figure 4C) displayed highly overlapping SNP sets with very few unique variants per strain. In contrast, *S. epidermidis* (Figure 4D) formed two major SNP intersection clusters with substantial divergence (up to tens of thousands of SNPs) and minimal overlap between clusters.

### 3.6 Antibiotic resistance gene (ARG) profiles and potential mobility across isolates

**Figure 5:**
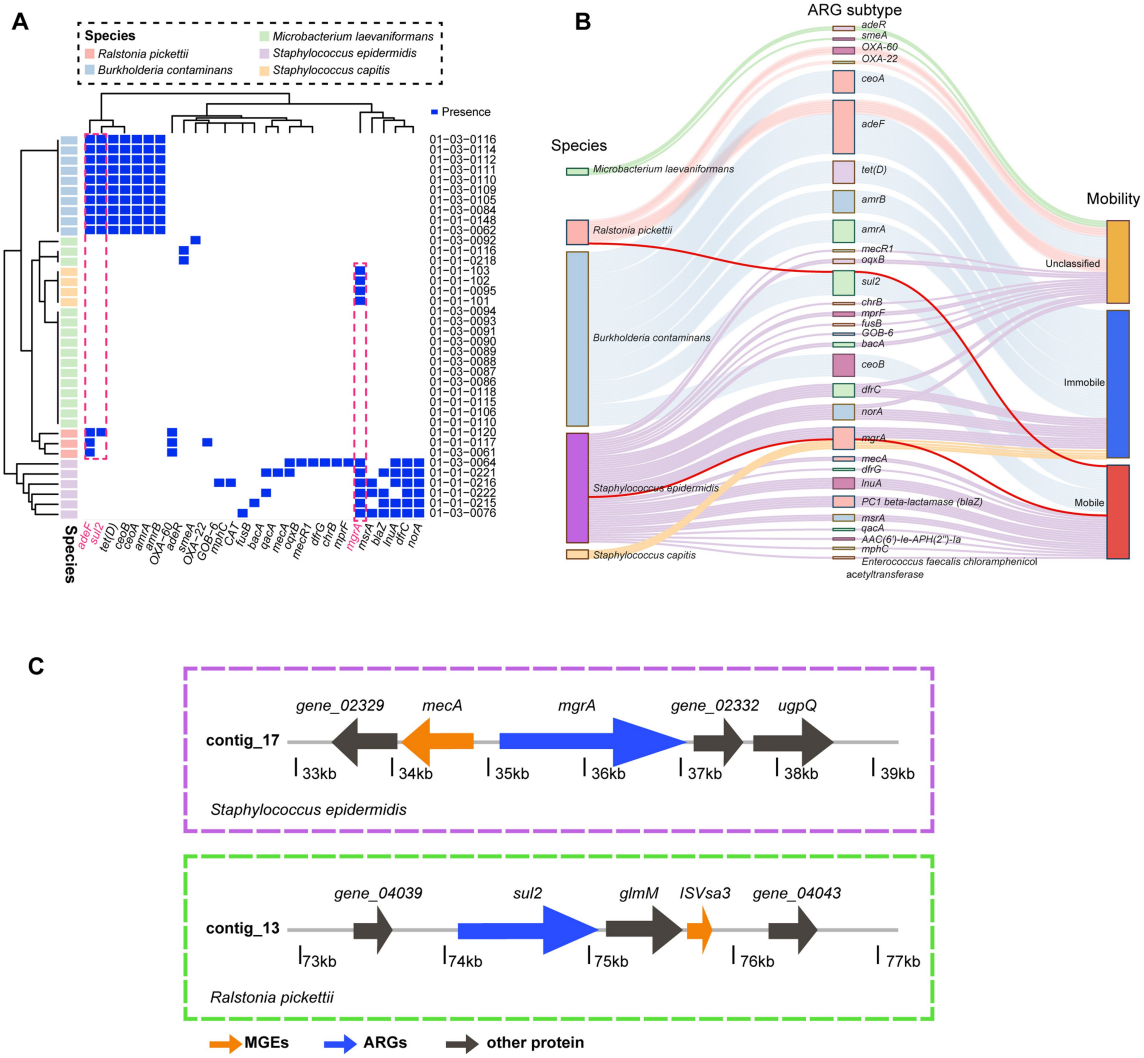
VFGs in environmental microbiomes of production facilities. **(A)** Comparison of ARG subtypes between isolates of five species. The boxes are colored blue if the subtypes was present in the sample (with identity ≥70%, alignment length ≥ of 25 amino acids, and *e*-value ≤ 10^−4^). **(B)** Sankey plot showing ARG subtypes and their mobility carried by five species form all samples. **(C)** Genetic background of *mgrA* genes in the *Staphylococcus epidermidis* and *Ralstonia pickettii*. The arrows indicate the direction of transcription of genes and are colored by the type of gene.

To further explore the resistome characteristics and potential horizontal gene transfer among isolates, we performed a comprehensive ARG survey based on whole-genome assemblies (Figure 5). The presence-absence heatmap (Figure 5A) revealed species-specific distributions of resistance determinants, In particular, *adeF* and *sul2* were simultaneously detected in *R. pickettii* and *B. contaminans*, while the regulatory gene *mgrA* was shared between *S. capitis* and *S. epidermidis*.

Given these interspecies ARG similarities, we further investigated their genomic contexts and mobility potential. Each contig containing ARGs was analyzed using PlasFlow for plasmid prediction, followed by manual classification according to established genomic location criteria:

(1) Mobile ARGs-those located on plasmids or flanked by MGEs within 2 kb; (2) Immobile ARGs-chromosome-borne genes without proximal MGEs; (3) Unclassified ARGs-genes on contigs of uncertain origin or lacking nearby MGEs.

The ARG presence-absence overview (Figure 5A) is retained as shown. In contrast, the mobility-resolved Sankey representation (Figure 5B) reveals a more nuanced pattern than previously described: all ARG subtypes detected within *M. laevaniformans* are classified as unclassified and show no shared subtypes with other taxa in our dataset, indicating an isolated resistome profile for this species in the sampled environment. By contrast, the *sul2* subtype is present in both *R. pickettii* and *B. contaminans*, and plasmid/contig-level analysis assigns this locus to the mobile category in *R. pickettii*. Similarly, the *mgrA* is found in *S. epidermidis* and co-occurs with the same subtype in *S. capitis*, with both occurrences classified as mobile.

### 3.7 Virulence factor distribution and prevalence

**Figure 6:**
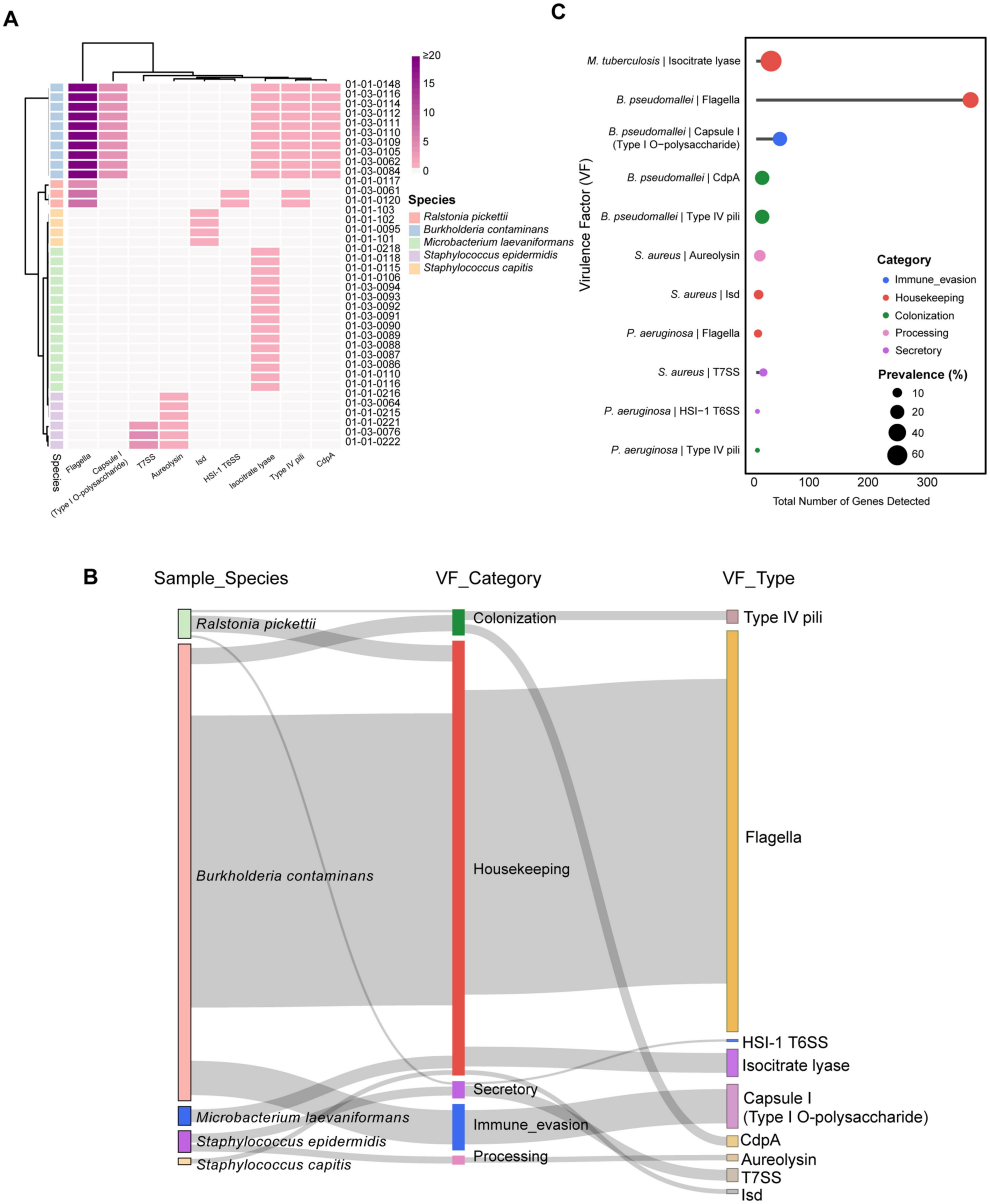
VFGs in environmental microbiomes of production facilities. **(A)** Comparison of VFGs between isolates of five species. The boxes are colored pure if the VFG was present in the isolate (with identity 90% and coverage = 70%), darker colors indicate higher VFG counts. **(B)** Sankey plot showing virulence factor category and virulence factor type form species of all samples. **(C)** The gene counts and prevalence of VFGs in 38 samples. The size of the circle represents the prevalence of each VFG, and the color of the circle represents the VF category. The *x*-axis represents the total number of each category.

Given that VFs are typically located within virulence islands, where individual genes may not function properly upon horizontal transfer due to the lack of associated regulatory elements, studying VF horizontal transfer presents significant challenges. As a result, we shifted our focus to analyzing the distribution of VFs to better assess microbial pathogenic risks in pharmaceutical production environments. This analysis allowed us to explore the distribution and overlap of VFs within the facility. The heatmap in Figure 6A illustrates significant variation in VF profiles across different species. For example, *B. contaminans* exhibited a broad spectrum of VFs, particularly those associated with immune evasion and colonization, such as Type IV pili. In contrast, *M. laevaniformans* displayed a much more limited VF repertoire.

The Sankey diagram (Figure 6B) further demonstrates the VF distribution across species. *R. pickettii* and *B. contaminans* primarily displayed VFs linked to the Colonization category, including Type IV pili and CdpA, highlighting their role in bacterial adherence. Conversely, *S. epidermidis* primarily linked to Secretory and Processing categories, while *B. contaminans* exhibits VFs associated with Immune evasion. *M. laevaniformans* and *S. capitis* exhibited VFs related to Housekeeping functions.

The bubble plot in Figure 6C highlights the prevalence of VFs across microbial species, with the size of the circles representing the prevalence of each VF within the sampled population. The most prevalent VFs were associated with colonization and housekeeping functions, with larger circles indicating their widespread presence across the isolates. For instance, we observed a high prevalence of immune evasion-related VFs across all samples (20.87%), with 40 detected genes, while certain Processing VFs were also relatively common (15.32%). This suggests that the pharmaceutical production environment harbors a considerable number of virulence factors. In summary, the virulence factor genes (VFGs) in the pharmaceutical production environment are primarily carried by opportunistic species. Colonization and housekeeping VFGs are the most prevalent, with *R. pickettii* and *B. contaminans* being the primary carriers. However, several VFGs were predominantly detected in *S. epidermidis*. These VFGs, including factors associated with secretion systems, may contribute to bacterial interactions with host cells and environmental adaptation under certain conditions.

## 4 Discussion

Despite stringent aseptic controls, microbial contamination persists in pharmaceutical sterile manufacturing, yet its genomic characteristics remain poorly understood. WGS provides the strain-level resolution required to elucidate contamination sources, dissemination pathways, and associated resistance and virulence risks. In this study, we applied WGS to comprehensively characterize microbial contamination in a sterile manufacturing environment, integrating species identification, transmission analysis, resistome profiling, and virulence assessment, thereby enhancing our understanding of microbial persistence and spread in pharmaceutical cleanrooms.

Pharmaceutical cleanrooms are designed to minimize microbial entry, yet contamination still occurs through multiple pathways associated with personnel, materials, and air handling (Whyte, 2010; Meng et al., 2024). Consistent with this, our findings showed that contamination primarily originated from raw materials and airborne routes, while personnel and surface-associated isolates also contributed notably to the contamination burden. Similar patterns have been reported in biopharmaceutical facilities, where inadequately protected supply chains and human-associated dispersal are recognized as major contamination drivers (Song et al., 2024). These results emphasize the necessity of stronger material transfer disinfection procedures and continuous personnel handling optimization to prevent microbial introduction.

WGS further enhanced our resolution of the environmental microbiota, confirming species-level clustering with ANI values >95%. The dominance of *B. contaminans* and *M. laevaniformans* suggests that certain organisms possess survival advantages enabling persistence in nutrient-limited cleanroom environments. *Burkholderia* species, in particular, have been shown to adapt to disinfectant-rich industrial ecosystems due to inherent stress resistance traits and biofilm-forming ability (Tavares et al., 2020). Meanwhile, *Staphylococcus* and *Ralstonia* isolates were primarily associated with downstream operations or personnel-related sampling points, consistent with prior evidence that these genera commonly originate from human skin or utility systems (Cogen et al., 2008; Ryan and Adley, 2014). These results demonstrate that microbial risks are not uniformly distributed across pharmaceutical facilities, but rather shaped by environmental function and exposure routes. Our findings also reinforce WGS as a more sensitive and informative tool than conventional testing for environmental surveillance, enabling early detection and lineage-based risk assessment (Baert et al., 2021; Song et al., 2024). Although ANI analysis revealed species-level relatedness and environmental associations, it cannot distinguish whether similar isolates reflect persistent survival or multiple reintroductions. SNP-level resolution is therefore essential to uncover contamination dynamics.

SNP-based comparative genomics provided deeper insight into the persistence and spread patterns of environmental isolates in the facility. The very small SNP variation in *B. contaminans*, *M. laevaniformans*, and *S. capitis* suggests that these strains originated from single clonal sources and underwent only minimal genetic changes during their persistence in the cleanroom environment (Tavares et al., 2020). The basal positioning of early isolates from Grade B areas suggested that *B. contaminans* likely originated from a single introduction followed by dissemination toward Grade A zones, while the personnel-derived basal isolate of *S. capitis* supports operator-associated transmission into surrounding airborne environments-consistent with its known colonization of skin-contact surfaces in aseptic manufacturing (Cogen et al., 2008; Whyte, 2010). In contrast, *S. epidermidis* showed profound SNP diversity, forming distinct lineages with minimal SNP sharing, demonstrating that this species is repeatedly reintroduced from human sources rather than maintained as a persistent clone (Otto, 2009; Castro et al., 2016). *R. pickettii* presented even greater divergence (>190 k SNPs), reinforcing utilities and water-contact systems as heterogeneous contamination inputs (Ryan et al., 2011).

These findings reveal that microbial persistence in pharmaceutical cleanrooms is species-specific: cleanroom-adapted opportunists undergo sustained persistence and microevolution, whereas human or utility-derived taxa enter through recurrent contamination events. It should be noted that these species-associated patterns were inferred from isolates collected within a single facility and a defined temporal and spatial sampling scope. Accordingly, the observed differences may reflect not only intrinsic biological traits of the species, but also the influence of sampling coverage, environmental niches surveyed, and operational conditions during the study period. In this context, the integration of SNP phylogeny and SNP-intersection profiling enabled high-resolution tracking of dissemination pathways across production units, informing tailored interventions-strengthening material-handling barriers for persistent clones and improving personnel and water-system control to curb repeated introductions.

Our resistome analysis showed that most isolates maintained species-specific ARG profiles, indicating that resistance patterns in this pharmaceutical cleanroom are primarily shaped by intrinsic genomic backgrounds rather than extensive interspecies exchange. However, the shared mobile *sul2* and *mgrA* subtypes across *R. pickettii*, *B. contaminans*, *S. capitis*, and *S. epidermidis*, together with their localization on plasmid-associated or MGE-flanked contigs, provide direct genomic evidence that sporadic horizontal transfer may occur even under stringent sanitation conditions (Bonvegna et al., 2022; Song et al., 2024). While the PlasFlow analysis allowed us to predict whether ARG-carrying and MGE-carrying contigs were plasmid or chromosome-derived, additional plasmid replicon typing would be useful in future studies to better assess potential plasmid dissemination among isolates. The persistence of such mobile elements in a highly regulated environment likely reflects multiple co-selection forces. Disinfectants and biocidal residues can promote plasmid retention and ARG-MGE stabilization (Wales and Davies, 2015) (Yu et al., 2016; Zhao et al., 2019), while biofilm formation on moist or water-contact surfaces facilitates DNA exchange and protects mobile determinants from removal (Flemming and Wingender, 2010). Regulatory elements such as MgrA may further enhance the persistence of mobile ARGs by coordinating stress responses and multidrug resistance pathways (Trotonda et al., 2009). Together, these mechanisms offer a plausible explanation for the episodic appearance of mobile resistance genes within ultra-clean facilities. Because such exchange events ultimately rely on horizontal gene transfer, it is necessary to consider its biological basis. Horizontal transfer is the exchange of genetic material within species without any sexual mechanism (Soucy et al., 2015). This phenomenon is widely documented in bacteria and has a role in bacterial evolution and adaptation (Soucy et al., 2015). This environment-dependent transfer pattern has been reported in a variety of environments. For instance, exposure to ARGs in coastal waters has been linked to an increase in ARG colonization in humans (Li et al., 2015), whereas exposure to ARGs present in aerosolized environmental samples in wastewater treatment plants (WWTP) correlate with increased abundance or diversity of ARGs in WWTP workers (Simar et al., 2021). These discrepancies suggest that the transfer and persistence of ARGs may be context-dependent, varying with environmental conditions, exposure routes, and the specific microbial community present.

The pronounced interspecies variability in virulence factor (VF) repertoires indicates that pathogenic potential within this pharmaceutical cleanroom is unevenly distributed. *B. contaminans* and *R. pickettii* exhibited enriched colonization and immune-evasion VFs, consistent with their documented ability to persist in moist industrial environments and contaminate sterile manufacturing systems (Ryan et al., 2011; De Volder et al., 2021). In contrast, the limited VF repertoire of *M. laevaniformans* suggests minimal pathogenic capacity, aligning with its characterization as a low-virulence environmental taxon (Wang et al., 2024). The detection of secretion-associated VFs such as T6SS and Aureolysin almost exclusively in *S. epidermidis* is notable, as these systems mediate interbacterial competition, epithelial invasion, and immune modulation (Otto, 2009; Ho et al., 2014). The presence of these virulence determinants highlights the ability of *S. epidermidis* to act as an opportunistic pathogen that may enter and persist in production systems when small lapses in aseptic procedures occur. Furthermore, the predominance of colonization and housekeeping VFs across species highlights selective pressures favoring adhesion, surface persistence, and biofilm-associated survival in cleanroom ecosystems-phenotypes repeatedly linked to contamination events and production interruptions in pharmaceutical facilities (Whyte, 2010). Taken together, the VF patterns indicate that microbial risks in this facility arise primarily from species with strong colonization and persistence capacities, whereas only a limited subset carries virulence systems directly associated with host interaction or tissue invasion. Integrating these VF signatures with resistome profiles allows high-resolution discrimination between environmentally persistent contaminants and strains with clinically relevant pathogenic traits, thereby refining microbial risk assessment in pharmaceutical production environments.

Integrating SNP phylogenies, sampling-location information, and ARG/VF distributions allowed us to reconstruct several plausible contamination trajectories within the facility. For *B. contaminans*, the core-SNP tree shows a tightly clustered clade with minimal genetic distances, and the earliest basal isolate originated from a Grade B environment, whereas genetically near-identical isolates were subsequently recovered from multiple Grade A rooms. This sampling-location pattern supports a single introduction in Grade B followed by inward clonal dissemination into Grade A (contamination chain: Grade B → Grade A). For *R. pickettii*, isolates separated into two distinct SNP-defined subclusters. One subcluster was exclusively associated with water-contact interfaces (e.g., sinks, taps), whereas the second appeared in Grade A/B areas without spatial or temporal continuity. These two genetically distant groups, together with their non-overlapping sampling sites, indicate repeated introductions from water-associated sources rather than within-facility spread (contamination chain: Water-contact interfaces → A/B cleanroom zones). For *S. epidermidis*, SNP distances were extremely large (6,000 ~ 27,000), forming several unrelated clusters mapped to personnel-contact surfaces (gloves, gowns, doors) distributed across nonadjacent rooms. The coexistence of multiple divergent lineages and their association with operator-touched surfaces indicate recurrent, personnel-mediated introductions rather than persistence of a single environmental clone (contamination chain: Personnel-contact surfaces → Multiple cleanroom rooms). For *M. laevaniformans*, isolates displayed the highest genomic divergence and were collected from unrelated equipment and locations (material-transfer bottle bottom, thermostatic water bath, simulation samples) without spatial or temporal linkage. This scattered pattern, together with the lack of any cohesive lineage, supports sporadic, low-level environmental entries without secondary spread inside the facility (contamination chain: External environment al or tempoth operator-touched).

Based on the findings of this study, we argue that WGS can substantially enhance the precision and scope of microbial source tracking in pharmaceutical manufacturing. Consistent with previous studies, WGS provides strain-level resolution that far surpasses traditional molecular typing approaches such as PFGE and MLST, enabling the accurate reconstruction of contamination sources and dissemination routes-capabilities increasingly recognized as essential in modern molecular epidemiology (Deng et al., 2016; Ruan et al., 2020). Beyond source attribution, genomic sequencing of antibiotic-resistant isolates offers significant advantages for resolving ARG hosts, mobility potential, and co-occurrence with virulence factors, thereby revealing mechanisms that underlie the persistence and spread of resistance genes within cleanroom environments. Our results highlight that pharmaceutical facilities may serve as hotspots for ARG-VF-MGE co-dissemination, driven by selective pressures imposed by disinfectants and antimicrobial agents and compounded by the possibility of horizontal transfer. Furthermore, personnel working in these environments are continuously exposed to resistant microorganisms, underscoring the need for routine genomic surveillance to detect the accumulation of resistance and virulence traits. Importantly, by integrating SNP phylogenies with spatial sampling information, WGS also enabled the inference of several plausible contamination chains within the facility, providing actionable insights into how different species enter and disseminate in cleanroom environments. Taken together, these insights illustrate the value of WGS as an actionable framework for real-time contamination control, proactive risk management, and the protection of both product quality and worker safety. As antimicrobial resistance continues to escalate globally, integrating WGS-based surveillance into pharmaceutical microbiology represents an increasingly critical step toward safeguarding sterile production systems.

## 5 Conclusion

Maintaining effective microbial control in pharmaceutical cleanrooms remains challenging because contaminants can enter and persist through multiple environmental and personnel-associated pathways. This study provides the genome-resolved characterization of microbial contamination in a pharmaceutical sterile manufacturing environment, integrating species identification, transmission inference, ARG profiles and VF assessment. The results show that microbial contamination is primarily driven by a limited number of opportunistic species, including *B. contaminans*, *M. laevaniformans*, and *S. capitis*, which exhibited extremely limited genomic diversity. Their tight SNP clustering and spatiotemporal distribution indicate persistence from either persistent environmental reservoirs or personnel-mediated transmission. By further combining SNP phylogenies with sampling location metadata and ARG/VF signatures, we inferred four plausible contamination chains that reflect distinct introduction routes and ecological behaviors within the cleanroom. Together, these findings underscore the superior resolving power of WGS over traditional typing methods and demonstrate its value as an actionable framework for source attribution, real-time contamination control, and proactive microbial-risk management. As antimicrobial resistance pressures and manufacturing complexity continue to rise, integrating WGS-based surveillance into routine pharmaceutical microbiology will be essential to safeguard both product quality and worker safety.

## Data Availability

The raw whole-genome sequencing data generated in this study have been deposited in the NCBI Sequence Read Archive (SRA) under accession number PRJNA1437902.

## Supplementary material

SUPPLEMENTARY TABLE S1 Summary of environmental sampling sites within sterile manufacturing facilities. The table lists the strains obtained from simulated purification procedures within the environmental monitoring program. For each isolate, the table provides detailed information on sampling source, cleanroom classification, Gram-staining characteristics, cellular morphology, MALDI-TOF or 16S rRNA identification results.

SUPPLEMENTARY TABLE S2 Summary of environmental abnormal sampling sites within sterile manufacturing facilities. The table lists 38 distinct sampling locations across key functional areas, including bulk liquid preparation, purification systems, cleanroom surfaces, utility systems, personnel contact sites, and airborne monitoring points, together with corresponding cleanroom classifications, and sampling positions.

SUPPLEMENTARY TABLE S3 Summary of whole-genome sequencing quality metrics for 38 microbial isolates. All samples achieved Q30 values above 95%, ensuring high base-calling accuracy and reliable sequencing depth for complete genomic coverage. The table provides detailed information on read number, GC content, coverage depth, and genome assembly statistics for each isolate.

SUPPLEMENTARY TABLE S4 Taxonomic assignment of 38 microbial isolates based on ANI analysis. A species delineation threshold of 95.0% ANI was applied following current genomic taxonomy criteria. The table lists ANI values between isolates and their closest reference genomes, together with corresponding species-level classifications and accession information.
