# Supplementary figures and images for: Leveraging whole-genome sequencing for microbial contamination tracking and risk assessment in pharmaceutical manufacturing
Source: Front Microbiol. 2026 Apr 15;17:1807989. doi: 10.3389/fmicb.2026.1807989 (PMC13125030; doi:10.3389/fmicb.2026.1807989)

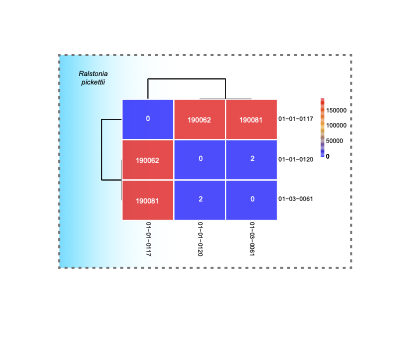

Supplement: Supplementary file 5 [file Figure_1.TIFF]
